# Impact of influenza vaccination programmes among the elderly population on primary care, Portugal, Spain and the Netherlands: 2015/16 to 2017/18 influenza seasons

**DOI:** 10.2807/1560-7917.ES.2019.24.45.1900268

**Published:** 2019-11-07

**Authors:** Ausenda Machado, Clara Mazagatos, Frederika Dijkstra, Irina Kislaya, Alin Gherasim, Scott A McDonald, Esther Kissling, Marta Valenciano, Adam Meijer, Mariëtte Hooiveld, Baltazar Nunes, Amparo Larrauri

**Affiliations:** 1 National Institute for Health Doutor Ricardo Jorge, Epidemiology department, Lisbon, Portugal; 2 These authors contributed equally; 3 NOVA National School of Public Health, Public Health Research Centre, Universidade NOVA de Lisboa, Lisbon, Portugal; 4 National Centre of Epidemiology, Carlos III Health Institute, CIBER of Epidemiology and Public Health (CIBERESP), Madrid, Spain; 5 National Institute for Public Health and the Environment (RIVM), Bilthoven, the Netherlands; 6 Epidemiology department, Epiconcept, Paris, France; 7 Nivel, Netherlands Institute for Health Services Research, Utrecht, the Netherlands

**Keywords:** influenza, influenza vaccine, impact, averted cases

## Abstract

**Background:**

To increase the acceptability of influenza vaccine, it is important to quantify the overall benefits of the vaccination programme.

**Aim:**

To assess the impact of influenza vaccination in Portugal, Spain and the Netherlands, we estimated the number of medically attended influenza-confirmed cases (MAICC) in primary care averted in the seasons 2015/16 to 2017/18 among those ≥ 65 years.

**Methods:**

We used an ecological approach to estimate vaccination impact. We compared the number of observed MAICC (n) to the estimated number that would have occurred without the vaccination programme (N). To estimate N, we used: (i) MAICC estimated from influenza surveillance systems, (ii) vaccine coverage, (iii) pooled (sub)type-specific influenza vaccine effectiveness estimates for seasons 2015/16 to 2017/18, weighted by the proportion of virus circulation in each season and country. We estimated the number of MAICC averted (NAE) and the prevented fraction (PF) by the vaccination programme.

**Results:**

The annual average of NAE in the population ≥ 65 years was 33, 58 and 204 MAICC per 100,000 in Portugal, Spain and the Netherlands, respectively. On average, influenza vaccination prevented 10.7%, 10.9% and 14.2% of potential influenza MAICC each season in these countries. The lowest PF was in 2016/17 (4.9–6.1%) with an NAE ranging from 24 to 69 per 100,000.

**Conclusions:**

Our results suggest that influenza vaccination programmes reduced a substantial number of MAICC. Together with studies on hospitalisations and deaths averted by influenza vaccination programmes, this will contribute to the evaluation of the impact of vaccination strategies and strengthen public health communication.

## Background

Influenza infections cause considerable morbidity and mortality [1,2], in particular among the elderly population 65 years and older. Vaccination is considered the most important public health intervention to reduce the incidence of seasonal influenza and its associated complications [3]. Following the World Health Organization recommendations [4], most European countries have a yearly influenza vaccination programme targeting, among others, individuals aged 65 years and older [5]. In most European Union (EU) countries, vaccine coverage (VC) in individuals in high-risk groups is below the target of 75% set by the EU Council [6].

To increase the acceptability of the influenza vaccine, it is important to assess the benefits of vaccination. Since 2008, influenza vaccine effectiveness (IVE) studies conducted every season in the EU suggest that the effectiveness of the vaccine is low to moderate in the elderly population [7,8]. These estimates provide useful information for scientists, but could be less comprehensible for the general public and for policymakers.

Estimating the impact of the influenza vaccination campaign focusing on the overall effect of the vaccine in the population has been an approach used by several countries [2,9-12]. Preaud et al. took a multi-country approach at European level [13]. In that study, the authors evaluated the public health and economic benefits of influenza vaccination, quantifying the prevented cases of influenza, hospitalisations and deaths in target groups for vaccination. They used country-specific data, when available, and interpolated data otherwise, that covered different seasons according to the different parameters. These authors quantified the differences in the number of influenza-confirmed associated events between the population exposed and the population not exposed to the vaccination programme using three parameters: IVE, VC and number of observed events. Impact was expressed as the number of influenza associated events averted by the influenza vaccination programme. These impact indicators to evaluate vaccine performance are easy to understand and to interpret.

To compare the number of averted events (NAE) in different countries with similar but not equivalent vaccination strategies, it is important to have not only a common approach but also harmonised parameter criteria. The lack of country-specific data limited the comparison of the impact of European influenza vaccination programmes [13]. Therefore, we developed a common protocol within the I-MOVE+ project [14] with a harmonised methodology that could measure, in different countries, the impact of the influenza vaccination programme. In this study, we aimed to assess the impact of the influenza vaccination programmes in Portugal, Spain and the Netherlands, by measuring the number of medically attended influenza-confirmed cases (MAICC) in primary care averted by vaccination, among the population aged 65 years and older, in three consecutive seasons (2015/16 to 2017/18). This was done by integrating existing estimates into new measures that may be more meaningful for public health policymakers and the public.

## Methods

### Study design

We developed an ecological study to estimate the number of medically attended influenza averted by the influenza vaccination programme.

We computed the number of averted events as:

$$NAE=n\times \frac{VC\times IVE}{1-\left(VC\times IVE\right)}$$

where *n* is the number of observed MAICC. To enable comparison between countries, NAE was also presented per 100,000 population. We estimated the prevented fraction as PF = NAE/(n + NAE). In addition, we calculated number needed to vaccinate (NNV) to prevent one MAICC, using methodology described in the Supplementary material.

### Input data

#### Influenza vaccination strategy

In the seasons 2015/16 to 2017/18, trivalent inactivated influenza vaccines were available for the population 65 years and older in the three countries. All countries had a national seasonal influenza vaccination programme in place and influenza vaccination was recommended for high-risk individuals (older age groups and individuals with chronic medical conditions) (Table 1). Seasonal influenza vaccination was recommended free of charge to individuals older than 60 years in the Netherlands, older than 60 or 65 years (depending on the region) in Spain, and 65 years and older in Portugal. 

**Table 1:** Country-specific influenza vaccination programmes and influenza surveillance systems, the Netherlands, Portugal and Spain, influenza seasons 2015/16–2017/18

Characteristics	Netherlands	Portugal	Spain
Vaccination programme			
Vaccine used	Inactivated trivalent vaccine		
Target population	**Population ≥ 60 years and medical risk groups (≥ 6 months-old)**	**Population ≥ 65 years and medical risk groups (≥ 6 months-old)**	Population ≥ 65 years and medical risk groups (≥ 6 months-old)
Payment	Free of charge for the target groups	Free of charge for those ≥ 65 years in public primary care units	Free of charge for target groups
Place of vaccination	Uptake via GPs	Uptake at pharmacy or healthcare units	Uptake mainly in public primary care units, but also in hospitals if needed, and in occupational risk units of public and private organisations
ILI surveillance system			
Surveillance network	Nivel Primary Care Database – Sentinel Practices	Rede Médicos-Sentinela	Spanish Influenza Sentinel Surveillance System
ILI case ascertainment and swabbing for confirmation	Weekly notification by sentinel GPs of cases meeting the ‘Pel-criteria’ [42]: sudden onset of symptoms AND fever (at least 38 °C) AND at least one of the following symptoms: cough, rhinorrhoea, sore throat, frontal headache, retrosternal pain or myalgia. GPs are recommended to swab at least the first two ILI patients attending each week.^a^	Weekly notification by sentinel GPs of cases meeting the EU ILI case definition	Weekly notification by sentinel GPs of cases meeting the EU ILI case definition [43]. GPs are recommended to swab the first two patients attending each week.
Laboratory confirmation of influenza	RT-PCR testing of nasopharyngeal/nose and throat swabs for influenza confirmation. If the test is positive for influenza, further tests are performed to determine virus type/subtype/lineage.		
Method of estimating positivity rate	Number of positive influenza detections among the swabbed respiratory samples × (1/sensitivity of RT-PCR); Sensitivity of RT-PCR = 0.95	Number of positive influenza detections among the swabbed respiratory samples	

EU: European Union; GP: general practitioner; ILI: influenza-like illness.

^a^ Patients < 65 years of age consulting on Monday through Wednesday and all ILI patients 65 years and older during the whole week.

#### Medically attended influenza-confirmed cases at primary care level

To estimate the number of observed MAICC, we combined epidemiological and virological data routinely collected by country-specific sentinel influenza surveillance systems (Table 1) during the surveillance epidemic period (week 40 to week 20). For Portugal, end-of-season cumulative ILI incidence rates in the seasons 2015/16 to 2017/18 were adjusted by end-of-season influenza positivity rate in the respective season and extrapolated to the national population aged 65 years and older. For Spain, weekly ILI and positivity rate were used to obtain ILI and number of positive cases. For the Netherlands, the observed number of MAICC was estimated using a component of an evidence synthesis modelling framework that integrates data on ILI incidence, influenza positivity rate and sensitivity of virological testing and is routinely used for estimating seasonal influenza incidence in the Netherlands [15]. Virus shedding peaks around 1 to 2 days after onset of symptoms, after which – for healthy persons – it usually declines to undetectable levels 7 days after onset of symptoms [15]. Therefore, we restricted the laboratory diagnostic data from the Netherlands to those patients diagnosed with ILI from whom a specimen had been collected not more than 7 days after symptom onset. For Spain and Portugal this restriction is not applied in the surveillance system. In both countries, the great majority of ILI patients recruited by sentinel general practitioners (GPs) were swabbed within the first 7 days after onset of symptoms (> 93%). ILI data were obtained from national sentinel GP networks (the Dutch Nivel Primary Care Database, the Spanish Influenza Sentinel Surveillance System (SISSS) and the Portuguese *Rede Médicos-Sentinela* [16-18] (Table 1).

#### Vaccine coverage

In the Netherlands, influenza VC in the population aged 65 and older was estimated using pseudo-anonymised data from electronic medical files of GPs participating in the Nivel Primary Care Database [19]. The VC point estimate as well as 95% confidence intervals (CI) were computed using multilevel logistic regression, taking into account the clustering of patients in GP practices [19]. In Spain, VC in the population aged 65 and older was provided by the Spanish Ministry of Health, based on administrative data of the number of doses of influenza vaccine administered [20]. In Portugal, VC was estimated using data from the 2015/16 and 2016/17 waves of a population-based telephone survey among the non-institutionalised population in mainland Portugal [21].

#### Vaccine effectiveness

We used the IVE among those aged 65 years and older estimated in the I-MOVE+ multicentre primary care-based test-negative design case–control study [22-24]. We pooled the VE of three seasons (2015/16–2017/18) (Supplementary Table S1) and weighted the (sub)type-specific VE by the proportion of influenza (sub)type detected in primary care settings in each country.

#### Uncertainty estimation

To estimate the 95% CI for NAE and PF, we used a probabilistic Monte Carlo approach. We constructed empirical distributions for influenza-associated outcomes, positivity rate, IVE and VC and used the 2.5 and 97.5 percentiles of these empirical distributions to compute the 95% CI for NAE and PF. All analyses were performed using STATA software.

### Ethical statement

The study was based on aggregated data obtained from official statistics, influenza surveillance systems and epidemiological studies (IVE studies) with scientific protocols approved by the national ethical committees of the three involved countries. Given the ecological nature of the study, no additional ethical approval was required.

## Results

### Input data

#### Medically attended influenza-confirmed cases at primary care level

In seasons 2015/16 and 2016/17, ILI epidemics occurred in similar periods in the three countries. In the 2017/18 season, a longer ILI epidemic was observed in the Netherlands (Supplementary Figure S1).

In all countries, the largest proportion of viruses detected in the sentinel networks were influenza A(H1N1)pdm09 in 2015/16, influenza A(H3N2) in 2016/17 and influenza B in 2017/18 (Table 2). The highest number of MAICC occurred in 2016/17 and 2017/18.

**Table 2:** Number of ILI, positivity rate and proportion of influenza (sub)types, MAICC, VC and VE among those aged ≥ 65 years, Portugal, Spain and the Netherlands, influenza seasons 2015/16–2017/18

	2015/16		2016/17		2017/18	
	n or %	95% CI	n or %	95% CI	n or %	95% CI
Portugal						
ILI (n)	9,161	6,656–12,297	21,646	17,289–26,766	12,366	9,340–16,057
Positivity rate (%)	27.8	20.5–35.1	47.4	40.2–54.5	46.2	43.0–49.3
MAICC (n)	2,547	1,641–3,686	10,261	7,673–13,087	5,708	4,247–7,298
VC (%)	50.1	42.1–58.1	57.5	50.8–64.1	60.8	55.5–65.9
Subtype A(H1N1)pdm09 (%)	90.4		0.2		20.0	
Subtype A(H3N2) (%)	1.3		99.6		14.0	
Type B (%)	8.3		0.2		66.0	
IVE (%)	40.6	22.6–58.6	8.5	−10.9 to 27.9	23.8	10.9–36.8
Spain						
ILI (n)	53,534	49,994–57,199	82,602	78,086–87,249	102,839	97,785–107,959
Positivity rate (%)	41.8	36.4–47.2	47.8	42.8–52.8	60.5	56.1–64.8
MAICC (n)	22,349	19,146–25,611	39,422	34,874–44,206	62,113	56,900–67,575
VC^a^ (%)	56.1		55.5		55.7	
Subtype A(H1N1)pdm09 (%)	69.9		0.0		7.6	
Subtype A(H3N2) (%)	4.0		94.4		25.3	
Type B (%)	23.9		0.6		64.9	
IVE (%)	34.0	18.5–48.4	9.0	−10.8 to 27.8	20.0	8.8–30.5
Netherlands						
ILI (n)	73,250	63,890–83,290	86,700	76,530–97,650	96,300	86,120–106,900
Positivity rate^b^ (%)	34.9	26.3–44.8	38.5	28.6–49.7	67.2	58.2–77.0
MAICC (n)	25,900	15,510–37,740	33,760	20,570–48,840	65,120	48,100–80,770
VC (%)	66.5	59.3–73.1	62.9	56.1–69.2	60.4	53.9–66.5
Subtype A(H1N1)pdm09 (%)	73.5		1.8		2.8	
Subtype A(H3N2) (%)	0.0		92.9		18.3	
Type B (%)	26.5		5.4		78.9	
IVE (%)	37.1	21.7–52.5	9.7	−8.4 to 27.8	19.5	4.7–34.4

CI: confidence interval; ILI: influenza-like illness; IVE: influenza vaccine effectiveness; MAICC: medically attended influenza-confirmed cases; VC: vaccine coverage.

^a^ 95% CI not available. VC in Spain calculated from administrative data, dividing the number of administered vaccine doses by the population.

^b^ Adjusted for test sensitivity.

#### Vaccine coverage

In the study period, VC in the population aged 65 years and older ranged between 50.1% (Portugal) and 66.5% (the Netherlands) (Table 2). The VC increased in Portugal (from 50.1% in 2015/16 to 60.8% in 2017/18), decreased in the Netherlands (from 66.5% in 2015/16 to 60.4% in 2017/18) and remained similar in Spain.

#### Influenza vaccine effectiveness

The IVE estimates ranged between 34.0% and 40.6% in 2015/16, 8.5% and 9.7% in 2016/17, and 19.5% and 23.8% in 2017/18 (Table 2). In the 2016/17 season, when influenza A(H3N2) virus was circulating in all countries, the IVE among the population 65 years and older was notably lower compared with other seasons.

### Impact of influenza vaccination in the prevention of medically attended influenza-confirmed cases

#### Number of averted events

Among those aged 65 years and older, influenza vaccination prevented an average per season of 715 MAICC in Portugal, 5,042 in Spain, and 6,457 in the Netherlands (Table 3). In Portugal, Spain and the Netherlands, the NAE per 100,000 population 65 years and older was 30, 61 and 275 in the 2015/16 season, 24, 24 and 69 in the 2016/17 season and 44, 88 and 268 in the 2017/18 season, respectively. The three seasons’ NAE rate was 204 cases per 100,000 in the Netherlands, 58 cases per 100,000 in Spain and 33 cases per 100,000 in Portugal (Table 3).

**Table 3:** Seasonal average, number and rates of MAICC events averted among those aged ≥ 65 years, by season, Portugal, Spain and the Netherlands, influenza seasons 2015/16–2017/18

Country	Indicator	2015/16	2016/17^a^	2017/18	Average
Portugal	NAE (95% CI)	650 (265–1,162)	527 (−746 to 1,876)	967 (316–1,701)	715 (215–1,246)
	Rate NAE/10^5^ (95% CI)	30 (13–52)	24 (−35 to 85)	44 (15–77)	33 (9.9–57.3)
	PF in % (95% CI)	20.3 (10.7–28.4)	4.9 (−7.9 to 15.1)	14.5(5.4–21.9)	10.7 (3.3–16.4)
Spain	NAE (95% CI)	5,268 (2,453–8,224)	2,073 (−2,657 to 6,758)	7,787 (2,891–12,648)	5,042 (2,602–7,500)
	Rate NAE/10^5^ (95% CI)	61 (29–96)	24 (−30 to 78)	88 (33–143)	58 (30–86)
	PF in % (95%CI)	19.1 (10.1–26.4)	5.0 (−7.1 to 14.5)	11.1 (4.4–16.9)	10.9 (5.9–15.3)
Netherlands	NAE (95% CI)	8,483 (3,396–16,255)	2,194 (−2,141 to 7,524)	8,694 (1,158–17,487)	6,457 (2,310–12,013)
	Rate NAE/10^5^ (95% CI)	275 (110–527)	69 (−68 to 238)	268 (36–540)	204 (74–380)
	PF in % (95% CI)	24.7 (12.7–34.6)	6.1 (−6.7 to 16.8)	11.8 (1.8–20.3)	14.2 (5.2–21.5)

CI: confidence interval; MAICC: medically attended influenza-confirmed cases; NAE: Number of averted MAICC events; PF: prevented fraction.

^a^ Negative lower bounds of 95% CI for NAE, averted rate and PF in 2016/17 season reflect the uncertainty around the point estimates and should not be interpreted as ‘negative impact’.

Parameter point estimates from Table 2 were used to calculate the point estimates of NAE, averted rate and PF.

#### Prevented fraction and number needed to vaccinate

The seasonal average estimates of MAICC prevented fractions were similar for the three countries. The PF ranged between 19.1% and 24.7%, in the 2015/16 season, between 4.9% and 6.1% in 2016/17 and between 11.1% and 14.5% in 2017/18 (Table 3).

As expected, the number needed to vaccinate to prevent one MAICC followed the pattern observed for NAE, with the lowest NNV values for season 2017/18 and the highest for season 2016/17 in all countries (Supplementary Table S2).

## Discussion

Our results suggest that during the 2015/16 to 2017/18 seasons, the influenza vaccination programmes in Portugal, Spain and the Netherlands had a sustained and positive impact on primary care MAICC in the population aged 65 years and older. The influenza vaccination programmes prevented an annual average of 33–204 primary care MAICC per 100,000 and 10.7–14.2% of potential MAICC that would have occurred without vaccination programme.

The impact of the influenza vaccination programmes varied across the influenza seasons. We obtained MAICC prevented fractions in the 2015/16 season of 19.1–24.7%, comparable to a study conducted in the United States (US) in 2013 that reported an average prevented fraction of 18.4% over six seasons [11]. 

We observed the lowest NAE during the 2016/17 season, when influenza A(H3N2) dominated in all countries. Given that VC did not vary considerably in the three seasons, the main drivers for the differences in season-specific NAE would be the number of MAICC and the IVE estimates. Seasons with dominant influenza A(H3N2) circulation are reported to produce a high influenza burden in the elderly population [25], and often a limited IVE against subtype A(H3N2) [26,27]. In 2016/17, the IVE was below 10% in the three countries. Despite this low protection, our results suggest that influenza vaccination programmes averted 24, 24 and 69 primary care MAICC per 100,000 population among those aged 65 years and older in Portugal, Spain and the Netherlands, respectively. This is consistent with other studies where, even in seasons with low vaccine effectiveness, the vaccination programme was able to avert influenza consultations, hospitalisations and deaths [2,9,28]. Particularly for the influenza vaccine with often limited effectiveness [26,29], such a message might illustrate considerable vaccine impact at population level, even when the vaccine effect at individual level is suboptimal.

Also the prevented proportion of primary care MAICC was the lowest in the 2016/17 season, namely 4.9% in Portugal, 5.0% in Spain and 6.1% in the Netherlands. A similar low PF (7–11%) was estimated in the US for seasons with predominant influenza A(H3N2) circulation [30,31]. The NAE results also differed by country, with Spain and Portugal both showing lower estimates than the Netherlands. As NAE is a linear function of primary care MAICC, a large difference in MAICC across countries or across seasons will lead to a large NAE difference.

In this study, we estimated the MAICC using primary care surveillance data. Potential explanations for the observed differences could be (i) the influenza positivity rate, (ii) the methods of the surveillance system, e.g. the case definition used to recruit ILI cases from the health system and (iii) the healthcare seeking behaviour.

The percentage of positive influenza cases among all tested varies between seasons and between countries, depending mainly on the (sub)type of the circulating virus and the sentinel GPs’ swabbing practice. The positivity pattern in the three countries was similar, with the highest positivity rate in the 2017/18 season, when influenza B virus accounted for more than 65%. The opposite was observed in 2016/17, when almost all isolates were influenza A(H3N2). This subtype is more frequent among older adults in whom ILI rates are generally lower [32], which could explain a lower positivity rate. 

Differences among countries can be derived from different (sub)type distributions in the circulating virus and also from different real swabbing practices between countries, even if, as in our study, systematic swabbing is established in the three countries. Another source of differences is the correction of the Dutch positivity rate for the RT-PCR sensitivity. Given that the RT-PCR sensitivity rate used was 95%, this would represent a systematic relative increase of 5.3% (1/0.95) in the Dutch positivity rates. This small increase does not explain the different NAE rate between countries.

Differences in national surveillance protocols may also play a role, e.g. the ILI case definition: In the Netherlands, the ILI case definition requires a fever ≥ 38 °C, while the EU ILI case definition used in Spain and Portugal only requires ’fever or feverishness’. Fever may be associated with more severe illness and with a higher likelihood of healthcare use [33,34]. In addition, the identification of ILI patients and the selection of patients for swabbing rely on the GP’s criteria, which may be influenced by how influenza surveillance has been done historically in their country, regardless of the ILI case definition used. Another important factor contributing to the different MAICC is probably the healthcare seeking behaviour in the age group 65 years and older. The use of primary healthcare has been described to be the highest in the Netherlands among the three countries [35]. In Portugal, the general population often uses emergency rooms at hospitals to treat acute illness [36], while in Spain and the Netherlands, the GP is the first point of call for an influenza consultation [37,38].

This study has limitations. One is the approach used to measure the impact assuming no indirect effect, i.e. no herd protection conferred by the vaccinated population to the non-vaccinated population. Dynamic model simulations have demonstrated that the indirect effect may not be negligible, particularly regarding the effect of vaccinating children on the adult population [39,40]. A meta-analysis revealed that influenza vaccination in children may result in herd protection for the community-dwelling elderly population against influenza-associated mortality [41]. However, to observe this herd protection in the population aged 65 years and older, a minimum VC of 20% is needed in children [39]. In our study, the indirect effect may be small since there is no overall vaccination recommendation for the younger population in any of the three countries and VC in adults younger than 65 years is presumably low. In Portugal for instance, the VC in all three seasons was below 5% in the 0–15 year-olds and between 7% and 18% in adults younger than 65 years [21]. In the Netherlands VC was 10% for the total population aged 18–64 years in the 2017/18 season [19]. However, non-vaccinated elderly people may still benefit from vaccination of younger age groups, particularly in settings with high VC in those 65 years and older [39]. As such, the NAE estimated in this study may be underestimated and the real impact of the vaccination programme could be even higher.

Another component that is not accounted for in our approach is that part of the estimated impact outcome measures may be attributable to previously acquired immunity, either through vaccination or natural infection. Our method does not allow us to distinguish which proportion among the prevented fraction is due to previous immunity and which is due to the current seasonal vaccination.

Another limitation to be acknowledged are the different ILI definitions and sources to estimate the VC, where some countries used population-based surveys, others GP surveys or administrative registries. In Portugal and the Netherlands, only the VC of community-dwelling elderly population is captured.

In Spain, regional administrative registries were used to calculate the national influenza VC. In the majority of the regions, the registry includes vaccines administered in both the public and the private sector. Only a few small regions report only vaccines administered in the public sector, therefore, we expect that influenza VC used reflected the VC in the population.

The study has several noteworthy strengths. Firstly, we used population-based surveillance data, so that the study can be replicated in several seasons and the results generalised for the population. Secondly, the IVE estimates derived from European pooled estimates were specific to the influenza (sub)type and adjusted to virus circulation in the country. This procedure not only allows us to obtain more precise estimates but also increases the robustness of the NAE results. Finally, we used country-specific data for all three countries individually, with harmonised analytical methods and definitions, allowing direct inter-country comparison.

## Conclusion 

The development of the common protocol resulted in a comparable population-based indicator of the impact of influenza vaccination programmes in the three countries. This can benefit existing influenza surveillance systems which already capture the annual influenza burden through national surveillance as well as estimation of the IVE through the I-MOVE network. Furthermore, by including severe influenza outcomes in future impact estimations, such as hospitalisations and deaths related to influenza, we will be able to provide a comprehensive view of the annual burden of influenza-related morbidity and mortality averted by vaccination. 

These results are important to support public health communication aiming to increase VC in high-risk groups. Influenza vaccination programmes can gain impact by increasing VC and/or IVE. Quantifying the benefit of annual vaccinations through estimates of their impact may contribute to this public health challenge, which could be a key message to the general public and decision makers, particularly in seasons with low vaccine effectiveness.

## Supplementary Data

288000 Supplement
