# Supplementary material for: Impact of influenza vaccination programmes among the elderly population on primary care, Portugal, Spain and the Netherlands: 2015/16 to 2017/18 influenza seasons
Source: Euro Surveill. 2019 Nov 7;24(45):1900268. doi: 10.2807/1560-7917.ES.2019.24.45.1900268 (PMC6852314; doi:10.2807/1560-7917.ES.2019.24.45.1900268)
Supplement: Supplement [file 19-00268_MACHADO_Supplement.pdf]

Influenza like illness rate-seasons 2015/16 to 2017/18- Portugal, Spain and the Netherlands

This supplementary material is hosted by *Eurosurveillance* as supporting information alongside the article **Impact on primary care of influenza vaccination programmes among the elderly population in Portugal, Spain and the Netherlands: 2015/16 to 2017/2018 influenza seasons** on behalf of the authors who remain responsible for the accuracy and appropriateness of the content. The same standards for ethics, copyright, attributions and permissions as for the article apply. Supplements are not edited by *Eurosurveillance* and the journal is not responsible for the maintenance of any links or email addresses provided therein.

Supplementary Figure S1. Influenza Like Illness (ILI) rates among the population aged 65 years and older, in Portugal, Spain and the Netherlands in seasons 2015/16 to 2017/18

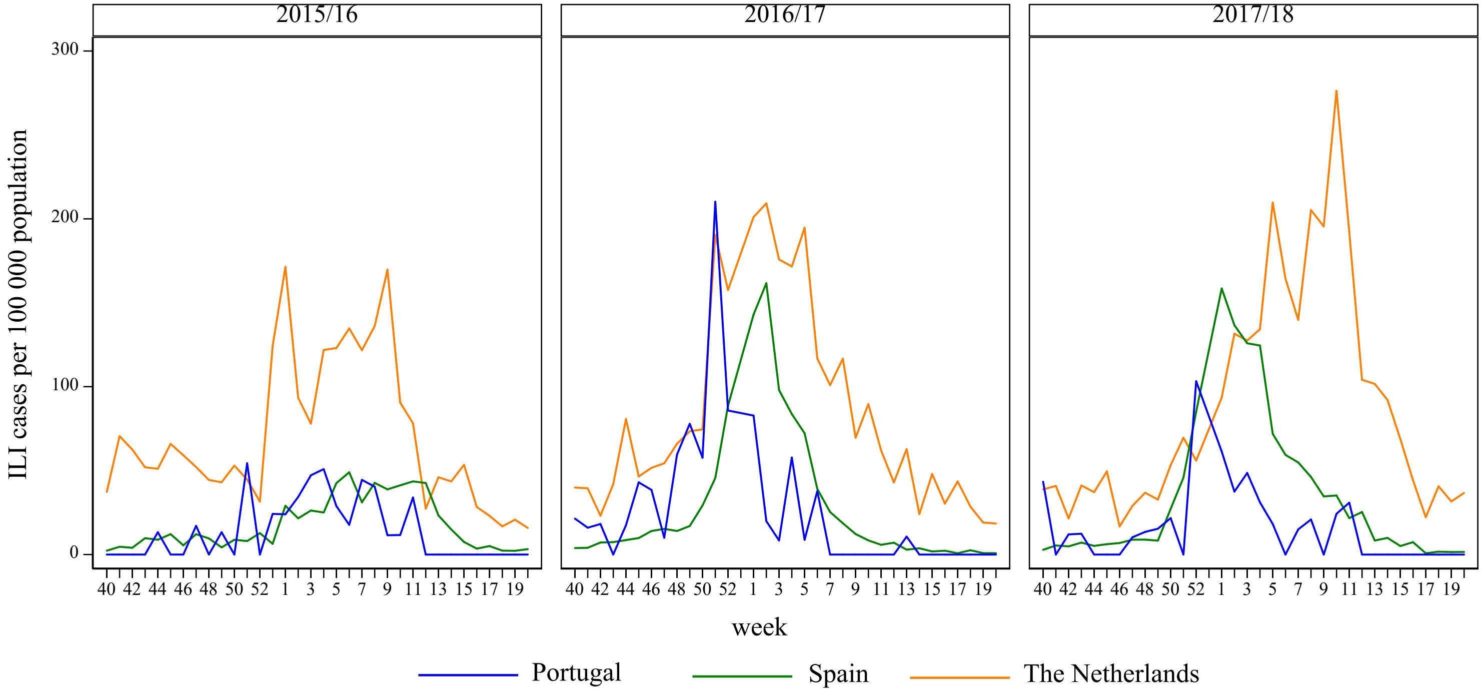

This supplementary material is hosted by *Eurosurveillance* as supporting information alongside the article **Impact on primary care of influenza vaccination programmes among the elderly population in Portugal, Spain and the Netherlands: 2015/16 to 2017/2018 influenza seasons** on behalf of the authors who remain responsible for the accuracy and appropriateness of the content. The same standards for ethics, copyright, attributions and permissions as for the article apply. Supplements are not edited by *Eurosurveillance* and the journal is not responsible for the maintenance of any links or email addresses provided therein.

Supplementary Table S1. Pooled type/subtyped IVE resulted from the I-MOVE+ multicenter primary care based study

**Type/subtype**	**Seasons included**	**IVE (95% CI)**
A(H1N1)pdm09	2015/16-2017/18	42.8 (19.6; 59.3)
A(H3N2)	2016/17-2017/18	8.4 (-13.1; 25.8)
B	2015/16-2017/18	21.3 (0.9; 37.5)

IVE: influenza vaccine effectiveness; 95% CI: 95% confidence interval

Number needed to vaccinate

This supplementary material is hosted by *Eurosurveillance* as supporting information alongside the article **Impact on primary care of influenza vaccination programmes among the elderly population in Portugal, Spain and the Netherlands: 2015/16 to 2017/2018 influenza seasons** on behalf of the authors who remain responsible for the accuracy and appropriateness of the content. The same standards for ethics, copyright, attributions and permissions as for the article apply. Supplements are not edited by *Eurosurveillance* and the journal is not responsible for the maintenance of any links or email addresses provided therein.

We estimated the number needed to vaccinate (NNV) to prevent one MAICC event as:

$$NNV = \frac{1}{IVE * (n + NAE) / Pop}$$

were IVE is the influenza vaccine effectiveness, n is the number of MAICC observed in the population in the presence of a vaccination programme, NAE is the number of MAICC averted by vaccination and Pop is the population size.
Confidence intervals for the NNV were computed incorporating uncertainty of each input parameter using Monte Carlo simulations. For the season when IVE 95%CI included the null value, the 95%CI for NNV are not provided.

NNV estimates are presented in Supplementary Table S2.

Supplementary Table S2. Number needed to vaccinate (NNV) to prevent one MAICC by season, in Portugal, Spain and the Netherlands in the elderly population. Influenza seasons 2015/16 to 2017/18

Country	2015/16 NNV(95%CI)	2016/17* NNV(95%CI)	2017/18 NNV(95%CI)
Portugal	1649 (964; 4057)	2374	1393 (774; 3839)
Spain	915 (586; 1963)	2333	632 (389; 1698)
Netherlands	242 (127; 598)	906	225 (105; 1093)

MAICC Medically-attended influenza confirmed case; 95% CI: 95% confidence interval

* For 2016/17 season the IVE estimate was not statistically significant (95% CIs for IVE included the null value) so the 95% confidence intervals for NNV are not provided
